# Avoidance of Interaction between Impression Materials and Tooth Surface Treated for Immediate Dentin Sealing: An In Vitro Study

**DOI:** 10.3390/ma12203454

**Published:** 2019-10-22

**Authors:** Bruna Sinjari, Gianmaria D’Addazio, Giovanna Murmura, Giorgio Di Vincenzo, Mario Semenza, Sergio Caputi, Tonino Traini

**Affiliations:** 1 Department of Medical Oral and Biotechnological Sciences, University G. d’Annunzio Chieti-Pescara, Via dei Vestini 31, 66100 Chieti (CH), Italy; gianmariad@gmail.com (G.D.); giovanna.murmura@unich.it (G.M.); scaputi@unich.it (S.C.); 2 Department of periodontics & implant dentistry, New York University, E 40th St #508, New York, NY 10016, USA; gtd2@nyu.edu; 3 Private practice, 26866 Sant’Angelo Lodigiano (LO), Italy; semenza.mario@gmail.com

**Keywords:** immediate dentin sealing, impression material, adhesive dentistry, prosthetic dentistry, in vitro

## Abstract

Immediate dentin sealing (IDS) is an advantageous approach for realizing adhesive restorations, but it interferes with the polymerization of impression material due to the oxygen-inhibition layer (OIL), which leaves residues of impression material on the teeth. The aim of this study is to identify a clinical surface cleaning protocol after IDS in order to achieve defect-free impressions. Sixty extracted human teeth were cut to expose fresh dentin and the IDS protocol was performed. Samples were divided into six groups where different surface cleaning protocols were made before taking impressions: G1S and G1P groups, IDS and silicone (S) or polyether (P) impressions; G2S and G2P, treatment with prophy paste and impressions; G3S and G3P, final cleaning phase with surfactant agent and impressions. Teeth were evaluated with a scanning electron microscope to identify the areas (expressed in mm^2^) where residual impression material was present. The results demonstrate a reduction of residues in the G2 groups and the total disappearance in G3 groups with statistically significant differences between them. Superficial cleaning protocols with the prophy paste and surfactant agent lead to the elimination of the interaction with impression materials and OIL. These results suggest a safe clinical protocol for obtaining defect-free impressions after IDS.

## 1. Introduction

In a population where patients’ lifespans have increased, the longevity of restorations is extremely important, together with an optimal functioning and aesthetics [1,2]. The possibilities of restoring compromised teeth have enormously increased over the years thanks to the use of a large number of materials and new technologies [3,4]. Each of these has advantages and disadvantages related to the intrinsic properties of the materials and their use protocols [5,6]. Regarding restorations supported by natural teeth, there are different possibilities ranging from fiber-reinforced composites to metal alloys and ceramic materials used for the manufacture of direct and indirect restorations such as inlays, onlays, veneers, and crowns [4,5,6]. The use of glass-ceramics and composite resins, due to their ability to adhere to enamel and dentin, allows minimally invasive preparation procedures. These procedures could guarantee a long life over the years [7]. 

The longevity of these restorations is guaranteed by correct adhesion procedures and by the quantity and quality of the residual tooth. This leads to a growing interest in dental research focused on ceramic restorations [8]. Among the disadvantages of the adhesive restoration materials is their vulnerability to gingival fluid, saliva and blood [9]. The contamination of restoration materials by these fluids during the adhesion step can lead to bacterial leakage, postoperative sensitivity, recurrent caries, discoloration, and failure of the restoration [10]. Thus, the control of contamination during the bonding procedure is a key factor in order to obtain a stable adhesion [11]. Due to the fact that dentin has a more heterogeneous substrate with higher organic and water content if compared with enamel, its bonding is really complex [8,9,10,11,12].

The traditional technique for indirect aesthetic restorations, the so called delayed dentin sealing technique (DDSt), consists in sealing dentinal tubules just before the positioning of the definitive restoration, and it has been the most utilized technique for sealing dentin tubules over the years. [13] Some negative aspects like the bacterial leakage, during the provisional restoration stage, risk of post cementation sensitivity, and incomplete seating of definitive restoration due to the thickness of dentin bonding agents layer, has been described [13,14,15,16]. During tooth preparation, exposure of dentin tubules is inevitable, and once they are opened they act like channels transmitting chemical, mechanical and bacterial stimuli to the pulp [17].

To avoid these problems in the early 1990s, a technique called immediate dentin sealing (IDS) was suggested, aimed at applying the adhesive directly after preparation of the tooth before taking the impression [18]. Over the years, this technique has been extensively studied, demonstrating how adhesive systems bind better to newly prepared dentin, thus protecting the dentin-pulp complex and increasing the adhesion strength [13]. A lot of advantages were described regarding IDS such as: bond strength improvement; stress free dentin bond development; pulp protection improvement and marginal/internal adaptation of indirect restoration; prevention of marginal infiltration and bacterial leakage, sensitivity reduction, promotion of patient comfort and tissue conservation [7,13,14,15,17,18,19]. IDS can also be useful for improving retention for short clinical crowns and excessive tapered preparations [20,21]. Finally, with IDS, clinicians can focus on the “wet bonding” to dentin while the bonding of dried enamel can be performed separately at the stage of definitive restoration placement [13].

Apparently, there are only advantages to using IDS compared with DDSt. On the other hand, problems have been described related to impressions on teeth treated with the IDS technique [14,22]. The impression material can interact with the outer resin layer. This external layer, called the oxygen-inhibition layer (OIL) appeared not polymerize, which may affect the impression procedure [14,22,23]. Several techniques have been described to reduce this layer, using alcohol or glycerin [14,22]. Magne et al. in 2009 [14] also investigated residues of the impression material visibly present on the tooth by optical microscope analysis, declaring that there is interference between teeth treated with IDS and the impression material. However, to the best of our knowledge, no study has provided clinical protocols for taking impressions after IDS by quantitative microscopic investigation. Therefore, the purpose of this work is to evaluate dental elements treated with IDS after taking impressions, following different clinical cleaning protocols. The null hypothesis is that there is no difference between an impression taken after IDS with and without the superficial cleaning procedure. 

## 2. Materials and Methods

The present study evaluated the teeth surfaces, treated with IDS, after use of different impression materials. A total of 60 periodontally compromised human teeth (25 molars, 25 premolars and 10 incisors) were extracted from 60 different patients from the clinic of the Department of Medical, Oral and Biotechnological Sciences, University of Chieti-Pescara, Chieti, Italy. All the extracted teeth were free of filling materials. 

**Figure 1:**
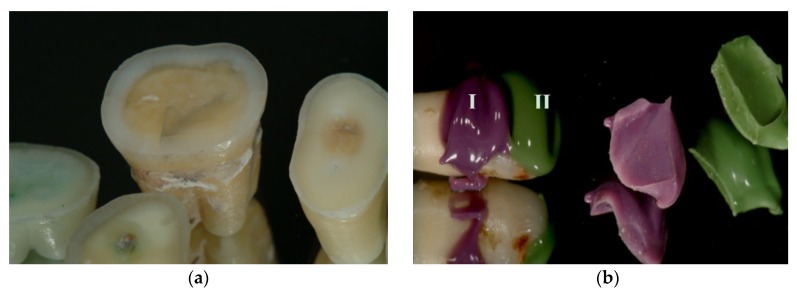
Image showing different teeth during preparation. (**a**) Teeth with exposed dentin, before immediate dentin sealing (IDS) treatment; (**b**) the same tooth sample, after IDS, which was impressed with polyether (I) and silicone (II).

Patients were informed that their extracted teeth were used for an in vitro study. To realize this study, a specific protocol was followed in order to obtain fresh cut dentin for the investigations. Specifically, the teeth were washed in running water, disinfected, and stored in distilled water. The first half coronal part of both molars and premolars or vestibular dentin of the incisors was removed with a diamond bur and later the created dentin surfaces were finished by means of grit paper under running water (Figure 1a). After removal of enamel all the specimens were treated with phosphoric acid at 37% for 30 s, cleaned with water spray for 20 s, slightly dried with air, and treated with Optibond FL (Kerr Corporation, Brea, CA, USA), polymerization was achieved by means of a light curing lamp of 800 mW/cm^2^ of power for 30 s with a Valo Cordless Ultradent (Valo cordless, South Jordan, UT, USA) using a light emitting diode (LED) with a wavelength ranging from 395 to 480 nm, using glycerin jelly to block air following the manufacturer instructions. Sixty extracted teeth were divided into six groups for the scanning electron microscope (SEM) analysis. 

The samples were divided into three groups (20 randomly chosen teeth per group) based on the type of cleaning procedure: Group 1 any surface treatment; Group 2 Prophy paste treatment (surface carefully cleaned with handpiece, coping brush and prophy paste (Detartrine z, Saint Maur des Fosses, France) at 500 rpm under water spray for 15 s) and Group 3 Prophy paste treatment and surfactant agent (Marseille soap) were used before impression procedures. Thus, all the teeth were treated in each half part with both impression materials; silicone (Extrude medium, Kerr Corporation) and the polyether (Impregum Penta; 3M ESPE, St. Paul, MN, USA) (Figure 1b) belonging to the following subgroups: 

G1S—any surface treatment after IDS and silicone impression; 

G1P—any surface treatment after IDS and polyether impression;

G2S—prophy paste treatment and silicone impression; 

G2P—prophy paste treatment and polyether impression; 

G3S—prophy paste treatment, cleaning with Marseille soap and silicone impression; 

G3P—prophy paste treatment, cleaning with Marseille soap and polyether impression.

The dental elements were then processed for scanning electron microscope (SEM) analysis according to the procedure previously discussed [24,25]. Summarily, using a SEM (Evo 50, Carl Zeiss, Oberkochen, Germany) it was possible to analyze the surface of each tooth. 

Metallization was done by a gold-sputter Emitech K550 (Emitech Ltd., Ashford, UK) and subsequently inserted into the sample-holder for SEM analysis. SEM operating conditions included 30 kV accelerating voltage, 10 mm working distance, and a 870 pA probe current. The images were captured with 20 scans using a line average technique.

The SEM images were displayed and studied using ImageJ software 1.48f 3D (Wayne Rasband at NIH), in order to highlight the areas of silicone and polyether linked on the surface in every sample. Statistical analysis was performed through a computerized statistical software using the SPSS (V. 24.0-IBM Corp., Armonk, NY, USA) and Excel (V. 15.39-Microsoft, Redmond, WA, USA) software. The results are presented as mean and standard deviation. The data were analyzed with descriptive statistics to assess whether they had a normal distribution. One-way analysis of variance (ANOVA) and Holm-Sidak tests were used to evaluate the overall significance and to perform all pairwise comparisons of the mean responses, respectively. A *p*-value of <0.05 was considered statistically significant. An independent statistician reviewed the methodology and statistical analysis done. 

## 3. Results

**Figure 2:**
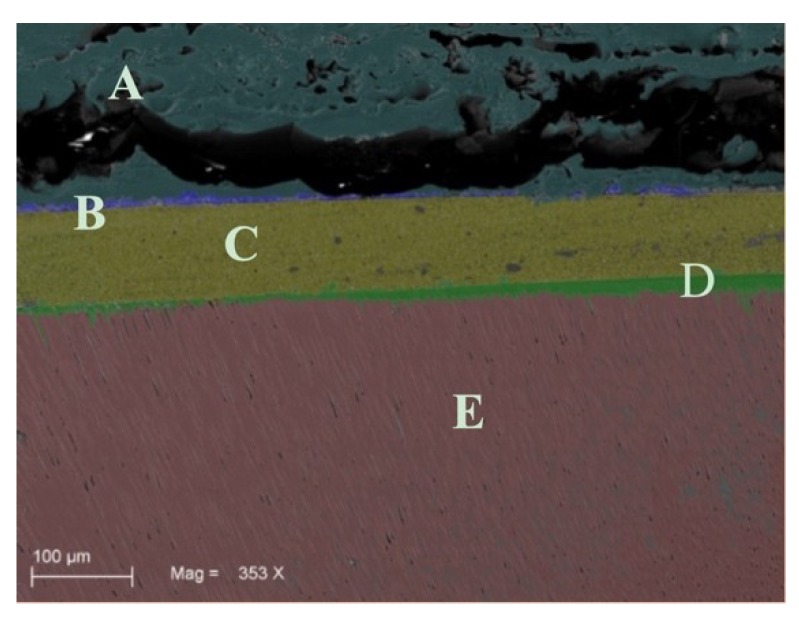
Image of Group 1. This image shows the interaction between the impression material and oxygen-inhibition layer (OIL). The letters indicate the different identifiable layers: (A) impression material; (B) interfering OIL layer with the impression material; (C) composite resin; (D) adhesive layer (it is possible to see the points where it penetrates into the tubules); (E) dentin.

**Figure 3:**
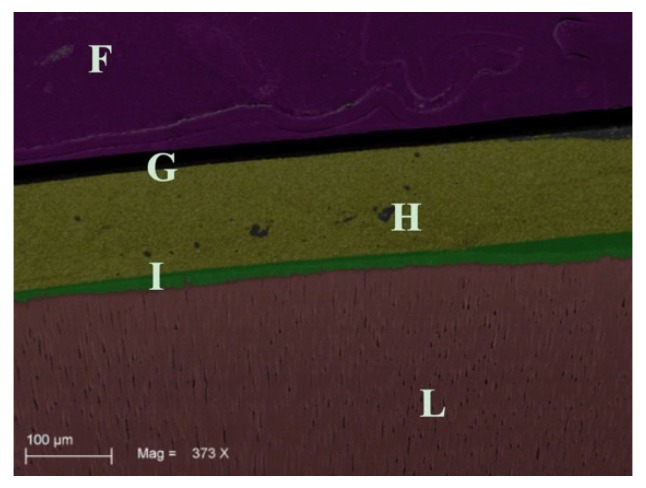
Image of Group 1. This image shows the interaction between the impression material and OIL. The letters indicate the different identifiable layers: (F) impression material; (G) no OIL was present on this image. A very regular layer of impression material was present near composite layer: (H) composite resin; (I) adhesive layer (it is possible to see the points where it penetrates into the tubules); (L) dentin.

**Figure 4:**
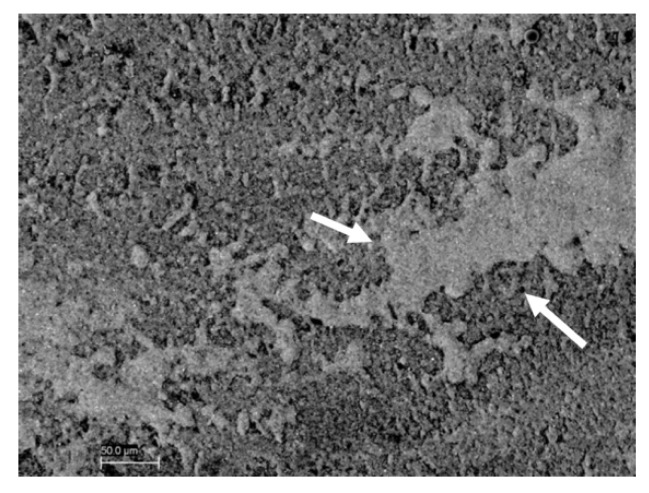
This image (Group 1) shows the interaction between the impression material and the OIL. It is possible to see the OIL on the tooth surface and the material impression bonds on it.

**Figure 5:**
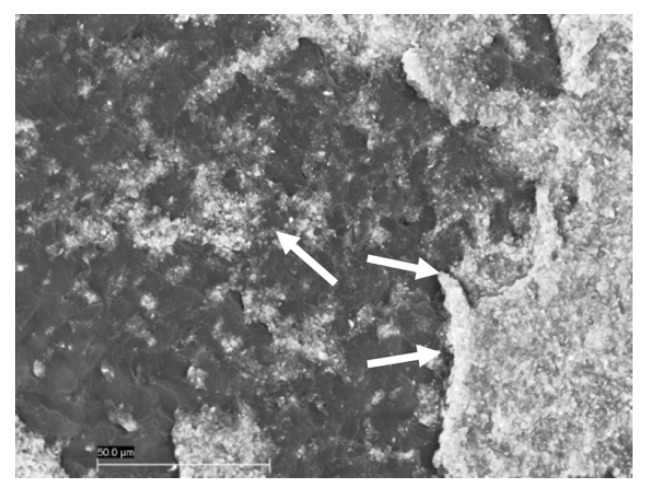
This image (Group 1) shows the interaction between the impression material and OIL. It is possible to see on the impression material the residual partial OIL removed with the impression.

**Figure 6:**
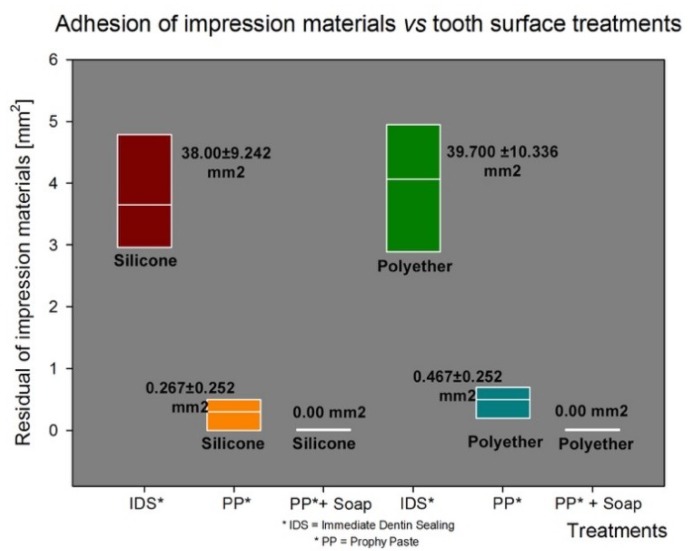
Graphical representation of residual of impression materials on teeth surfaces. The image could help to illustrate the difference between the cleaning protocols, but not between the impression materials used.

Sixty samples divided into six groups were treated and analyzed by SEM analysis. On the microscopic analysis, different degrees of contaminated dentin were found between groups. In fact, the presence of OIL, silicone, and polyether materials on dentin surface of groups G1S, G1P, G2S, and G2P were easily identifiable on SEM images (Figure 2). On the other hand, no residual impression material was found on teeth in any samples from Group G3S and G3P. In addition, no OIL was detectable on samples analyzed after the cleaning procedure was performed on groups G3S and G3P (Figure 3). Specifically, the areas of silicone and polyether materials linked on the surface in the groups were 38.00 ± 9.242 mm^2^ and 39.700 ± 10.336 mm^2^ respectively for the G1S and G1P groups. Meanwhile, in the Group 2 the mean areas were 0.267 ± 0.252 mm^2^ and 0.467 ± 0.252 mm^2^ respectively for group G2S and Group G2P. Finally, in the group G3S were 0.00 mm^2^ and G3P were 0.00 mm^2^. Only the teeth from Group G3S and G3P showed no residual impression material on their surface as shown in Figure 3. Moreover, in Figure 4 and Figure 5 it is possible to see the residual impression materials on teeth (Figure 4) and part of the residual OIL on impression taken (Figure 5). The statistical analysis of variance (ANOVA) showed a statistically significant difference between groups, confirming the SEM results, as shown in Table 1 (*p* < 0.05). Figure 6 demonstrates a graphical representation of residual impression material expressed in mm^2^ on teeth, into the six subgroups analysed (Figure 6). Furthermore, analyzing the individual treatments, all pairwise multiple comparison procedures (Holm-Sidak method) between the subgroups showed statistically significant difference as shown in detail in Table 1. 

## 4. Discussion

The null hypothesis of this study was rejected, demonstrating that a defect-free impression could be achieved by different cleaning procedures on teeth after IDS. 

Certainly, the undoubted advantages of the IDS protocol but also the disadvantages linked to the presence of IDS, have been widely described in the literature. Among the disadvantages, an excessive residual thickness of the adhesive layer and consequently possible interferences during the positioning of the restoration, or the possible interference with impression material, have already been described [7,13,14,15,22]. The results of the present study revealed, by SEM analysis, a large quantity of impression material residues on teeth after IDS treatment. These residues were present in both groups where the polyether and silicone were used. The presence of the OIL layer could probably be the cause of the surface alterations. This layer can represent up to 40 μm of external surface that does not polymerize in contact with oxygen [22,23]. Previous studies demonstrated that the superficial presence of residual monomers can create interaction with impression materials. In fact, in common adhesive systems and composite resins, composed of methacrylates, after photopolymerization, free monomers remained on the surface resulting in continue reaction with common impression materials [14,22].

The present results demonstrate that in the samples where the prophy paste treatment was carried out (G2), the amount of residue of impression material, although present, was lower than in other subgroups. Statistically significant differences were present between groups G1 and G2 and G1 and G3, for both impression materials. Therefore, the use of prophy paste treatment has shown to significantly reduce the interaction between surface monomers and impression materials. The use of a surfactant agent (Marseille soap) introduced in the third group, allowed for the complete elimination of the presence of surface residues in all the treated samples. It was demonstrated by these results, how the use of Marseille soap, after prophy paste treatment, is an essential step for the removal of the superficial OIL and therefore necessary to fully enjoy the advantages of IDS. Other authors in the literature have emphasized the problem of the adhesion between OIL and impression material following IDS [14,22]. Specifically, Magne et al. in 2009 [14] already demonstrated this aspect of impression material after IDS. The authors concluded that residues on the teeth were present and could be reduced with the air blocking and pumice protocol, obtaining defect-free impressions, but only using silicone. However, defects were present with polyether [14]. By the way, only an optical light microscopy observational analysis on six human extracted teeth was performed. On the contrary, a quantitative analysis performed on SEM images on 60 samples was done here. In our work, the air blocking was in any case carried out after IDS, as recommended by the manufacturer. However, this was not sufficient, as shown by SEM images (Figure 2), to obtain defect-free impressions. The SEM analysis made it possible to carry out a thorough investigation that showed the microscopic residues present in all the samples treated only with air-blocking. As demonstrated by the SEM images, it was possible to note residues of impression materials linked to the superficial layer of the tooth and, on the contrary, the OIL layer inside the impression after its removal. In the qualitative study of Magne, no visible residues emerged with a silicon impression after air blocking protocol. The authors concluded that interactions were still present in the impression taken with polyether by discouraging this material in clinical use after IDS [14]. On the contrary, our results, following treatment with surfactants allowed us to conclude that the residues can be totally eliminated and that both materials can be used after IDS, presenting perfect and defect-free impressions. Another study was focused on the interaction between OIL and impression materials [22]. Ghigghi et al. in 2014 [22], photographed 35 extracted human teeth showing in the control group (no IDS) that there were no visible residues of impression material. On the contrary, in the group with IDS treatment both materials (silicone and polyether) had defects. Specifically, the silicone materials showed an incomplete polymerization and the polyethers, although polymerized, attempted to adhere to the resin present on surface. In the test group (treated with glycerin jelly and alcohol test) samples showed a reduced OIL, allowing fir an impression without defects. Moreover, in this study, the authors carried out only a qualitative and visual evaluation on the surface of the teeth treated [22]. This could be a limit of the study because without microscopic images, surface defects may be difficult to detect. On the contrary, our samples, analyzed by SEM, showed how the simple air blocking with glycerin does not allow for the elimination of surface interactions with free monomers. 

For this reason, a more effective surface cleaning protocol is required that allows to take a defect-free impression with both materials. Inadequate impressions did not allow to realize a precise dental restoration with an adequate marginal adaptation [26]. Studies showed that the technological increase in impression materials let to obtain very detailed impression and the most visible errors were attributable to the techniques used rather than to the material itself [27,28]. In this sense, a precise and repeatable clinical protocol for cleaning a tooth surface can reinforce the advantages of IDS techniques together with high quality and precision impressions. Authors also demonstrated different results depending on the type of adhesive used during IDS [14,22,29,30,31]. Specifically, Magne demonstrated an adhesive with a lower viscosity had a thinner OIL layer that could be virtually eliminated as a result of air blocking and pumice. Specifically, the comparison presented was between the SE bond and Optibond SL, in favor of the former [14]. Our samples, despite having all been treated with the Optibond adhesive, and therefore being more loaded, showed no interactions following the surface cleaning treatment. However, future research should be performed in order to analyse in vivo fresh cut dentin, during the clinical procedure. 

## 5. Conclusions

Within the limitations of the present study, the following conclusions were drawn. The cleaning protocol has the greatest influence on the residual impression material. Prophy paste and Marseille soap represent the best way to overcome the interaction between impression materials and IDS tooth surfaces. The correct clinical protocol in order to create an ideal impression giving no limitation to the clinician regarding the use of impressions materials and IDS technique was found.

## Figures and Tables

**Table 1:** This table shows statistically significant difference between groups (*p* < 0.01) using one-way analysis of variance. In the second part, the Holm-Sidak method was performed to verify the significance of the difference between every single subgroup. Only between G2 and G3 are there no statistically significant differences.

One Way Analysis of Variance					
Group Name	N	Missing	Mean	Std. Dev.	SEM
G1S	20	0	38	9.242	2.067
G1P	20	0	39.7	10.336	2.311
G2S	20	0	0.267	0.252	0.0563
G2P	20	0	0.467	0.376	0.0841
G3S	20	0	0	0	0
G3P	20	0	0	0	0
**Source of Variation**	**DF**	**SS**	**MS**	**F**	***p***
Between Groups	5	39,901.28	7980.256	248.797	<0.001
Residual	114	3656.594	32.075		
Total	119	43,557.874			
**Comparisons for Factor:**					
**Comparison**	**Diff. of Means**	**t**	**Unadjusted P**	**Critical Level**	**Significant**
G1P vs. G3P	39.7	22.167	3.849 × 10^−28^	0.003	Yes
G1P vs. G3S	39.7	22.167	3.849 × 10^−40^	0.004	Yes
G1P vs. G2S	39.433	22.018	7.19 × 10^−43^	0.004	Yes
G1P vs. G2P	39.233	21.906	1.15 × 10^−42^	0.004	Yes
G1S vs. G3S	38	21.218	2.153 × 10^−38^	0.005	Yes
G1S vs. G3P	38	21.218	2.153 × 10^−38^	0.005	Yes
G1S vs. G2S	37.733	21.069	4.09 × 10^−41^	0.006	Yes
G1S vs. G2P	37.533	20.957	6.627 × 10^−38^	0.006	Yes
G1P vs. G1S	1.7	0.949	0.345	0.007	No
G2P vs. G3S	0.467	0.261	0.795	0.009	No
G2P vs. G3P	0.467	0.261	0.795	0.01	No
G2S vs. G3P	0.267	0.149	0.882	0.013	No
G2S vs. G3S	0.267	0.149	0.882	0.017	No
G2P vs. G2S	0.2	0.112	0.911	0.025	No
G3S vs. G3P	0	0	1	0.05	No

The differences in the mean values among the treatment groups are greater than would be expected by chance; there is a statistically significant difference (*p* ≤ 0.001). Power of performed test with alpha = 0.050:1.000. All Pairwise Multiple Comparison Procedures (Holm-Sidak method): Overall significance level = 0.05.
